# Novel platform technology for modular mucosal vaccine that protects against streptococcus

**DOI:** 10.1038/srep39274

**Published:** 2016-12-15

**Authors:** Mehfuz Zaman, Victoria Ozberk, Emma L. Langshaw, Virginia McPhun, Jessica L. Powell, Zachary N. Phillips, Mei Fong Ho, Ainslie Calcutt, Michael R. Batzloff, Istvan Toth, Geoffrey R. Hill, Manisha Pandey, Michael F. Good

**Affiliations:** 1 Institute for Glycomics, Griffith University, Gold Coast, QLD 4222, Australia; 2 The University of Queensland, School of Chemistry and Molecular Biosciences, St Lucia, QLD 4072, Australia; 3 The University of Queensland, School of Pharmacy, Woolloongabba, QLD 4102, Australia; 4 Institute for Molecular Biosciences, The University of Queensland, St Lucia, QLD 4072, Australia; 5 QIMR Berghofer Medical Research Institute, QIMR Berghofer Centre for Immunotherapy and Vaccine Development, Brisbane QLD 4029, Australia; 6 Bone Marrow Transplant Unit, Royal Brisbane Hospital, Brisbane, QLD 4006, Australia

## Abstract

The upper respiratory tract (URT) is the major entry site for human pathogens and strategies to activate this network could lead to new vaccines capable of preventing infection with many pathogens. Group A streptococcus (GAS) infections, causing rheumatic fever, rheumatic heart disease, and invasive disease, are responsible for substantial morbidity and mortality. We describe an innovative vaccine strategy to induce mucosal antibodies of significant magnitude against peptide antigens of GAS using a novel biocompatible liposomal platform technology. The approach is to encapsulate free diphtheria toxoid (DT), a standard vaccine antigen, within liposomes as a source of helper T-cell stimulation while lipidated peptide targets for B-cells are separately displayed on the liposome surface. As DT is not physically conjugated to the peptide, it is possible to develop modular epitopic constructs that simultaneously activate IgA-producing B-cells of different and complementary specificity and function that together neutralize distinct virulence factors. An inflammatory cellular immune response is also induced. The immune response provides profound protection against streptococcal infection in the URT. The study describes a new vaccine platform for humoral and cellular immunity applicable to the development of vaccines against multiple mucosal pathogens.

The respiratory tract is the most common site of infection by pathogens. Upper respiratory tract (URT) infections cause at least half of all illness in the community, resulting in morbidity, absenteeism from work and school, direct health care costs and mortality^1^. A wide range of organisms infect the respiratory tract, including viruses, bacteria, fungi and parasites. Group A streptococcus (GAS) is a Gram-positive bacterium that primarily infects the URT mucosa, but also the skin, resulting in a broad spectrum of diseases. The most common disease following colonization of the URT is pharyngitis. However, there are several ‘post-streptococcal’ diseases that are of particular concern, including rheumatic fever and rheumatic heart disease (RHD), for which there is an estimated 34 million prevalent cases^2^. Globally, GAS infection and associated diseases result in over 500,000 deaths each year, highlighting the urgent need for a vaccine.

GAS vaccines can be divided into M-protein and non-M protein-based candidates^3^. The M protein is a major virulence determinant located on the cell surface^4^ and consists of a hypervariable amino-terminus that is the target of strain-specific antibodies and a conserved C-repeat domain. Leading M protein-based vaccine candidates are the amino-terminal multivalent vaccines and conserved C-repeat M-protein peptides^5^,^6^,^7^. Non-M protein candidates include SpyCEP^8^, C5a peptidase^9^ and group carbohydrates^10^.

We previously defined two conserved vaccine peptides: J8-based on a minimal B cell epitope from the C3-repeat domain of the M protein^11^; and S2, from the bacterial IL-8 protease, SpyCEP^12^. When chemically linked to the carrier protein diphtheria toxoid (J8-DT), and administered sub-cutaneously with alum, J8 induces IgG antibodies that together with neutrophils protect mice from systemic and skin challenge with multiple GAS strains^6^,^13^. S2 is a 20-amino acid peptide from SpyCEP^12^. Immunization with S2-DT induces antibodies that can block the proteolytic function of SpyCEP and protect the neutrophil chemo-attractant activity of IL-8. The combination of J8-DT with S2-DT, administered parenterally with alum, induces IgG antibodies that can protect against skin challenge with hyper-virulent GAS organisms that have mutated their Control of Virulence Regulatory Sensor (CovR/S) and thus up-regulated SpyCEP production^12^.

Unlike protection against skin disease, protection against URT infection requires an IgA response. Passively acquired human M-protein-specific IgA, but not IgG, can protect mice from intranasal infection^14^, and mucosal immunity in mice following immunization with a proteosome-based vaccine correlated with a salivary IgA response to a conserved M-protein peptide^15^. However, IgA production requires mucosal immunization and there are no mucosal adjuvants licensed for human use^16^, although some are highly effective in inducing mucosal IgA responses in animals (such as the cholera toxin B [CTB] subunit) following intra-nasal administration^17^.

The aim of this study was to develop a novel vaccine formulation that would induce mucosal immunity and be suitable for human use. The natural tendency of liposomes to interact with antigen presenting cells combined with the known ability of lipid core peptides to target Toll Like Receptors^18^,^19^ provided the rationale for using liposomes combined with lipidated peptides to activate the immune system^20^. Various liposome formulations have been administered safely to humans^21^.

Liposomes are spherical vesicles composed of biocompatible phospholipid bilayers^22^ in which multiple lipophilic constructs can be incorporated within the lipid bilayers. They should be ideal platforms to induce peptide-specific antibody responses of multiple specificities. However, peptides are poorly immunogenic unless conjugated to a ‘carrier’ protein that activates helper T-cells^23^. The classic strategy has been to conjugate peptides to carrier proteins or to T-helper epitopes before embedding them in liposomes^24^. This was based on fundamental immunological principles whereby B-cells recognizing the B-cell epitope take up the linked protein or T-cell epitope which is then processed for presentation to T-cells^23^. Thus, to induce antibodies of multiple defined specificities with liposomes, each peptide will require chemical conjugation prior to liposome assembly and the capacity to present multiple epitopes will depend on the choice of carrier protein. There are no peptide-liposome vaccines in clinical trial (www.clinicaltrials.gov). The question that we addressed was whether a carrier protein contained within a liposome, but not physically conjugated to the peptide, would be able to induce T-cell help to B-cells responding to different surface peptide epitopes of GAS and induce protection against this mucosal pathogen. If successful, such liposomes would transform vaccine development by allowing modular design of multiple peptide immunogens effective against many mucosal pathogens.

## Results

Parenteral immunization with J8-DT/alum induces J8-specific antibodies and protects against skin and systemic infection with common strains of GAS of multiple *emm* types via IgG antibodies working together with neutrophils^13^. We initially asked whether this vaccination regimen would protect against URT infection. Mice were vaccinated with three doses of J8-DT/alum intra-muscularly following which we observed high serum IgG titers (10^5^–10^6^) (Supplemental Fig. 1A), serum IgA titers of 100 (data not shown), salivary IgG titers of ~10 (data not shown) and no salivary IgA (Supplemental Fig. 1B). Mice were then challenged with the M1 GAS strain (in two separate experiments) and we observed limited reduction in GAS colonies from nasal secretions, in throat, and in Nasal Associated Lymphoid Tissue (NALT; a murine functional homolog to human tonsils)^25^, which did not reach statistical significance (Supplemental Fig. 1C–H).

These data provided the impetus to develop a mucosal vaccine platform that could induce peptide-specific IgA and protection of the URT compartments. Given our evidence that protection against the highly virulent CovR/S mutant GAS required the induction of antibodies capable of neutralizing two complementary virulence factors^12^, we developed a modular vaccine that could present more than one peptide simultaneously.

### Liposome vaccine immunogenicity and stability

**Figure 1:**
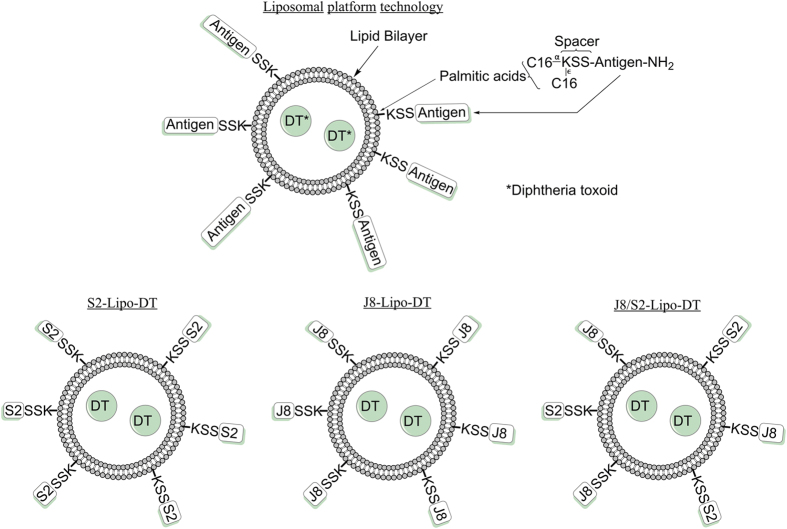
Idealized structure of liposomal platform technology. Liposome encapsulates DT while the peptide antigens attached to the spacer KSS at the N-terminus is covalently coupled to two palmitic acid molecules, facilitating their insertion into the liposome membrane (both internally and externally). For simplicity, the lipidated peptides are only displayed as being external to the liposome, but are expected to be located on both sides of the membrane.

The vaccine platform that we tested consisted of a liposome composed of neutral lipids encapsulating DT and displaying various lipidated charged peptides on its surface. The vaccine is referred to as ‘Peptide-Lipo-DT’. The peptides represented target B-cell epitopes with the lipid tail anchoring the peptide to the liposome. The liposomes contained an average of 57 peptides per molecule of DT (total DT content was 87 μg encapsulated within 10 mg of liposomes). Ninety-five percent of the DT was encapsulated within the liposome as determined by using a fluorescent analogue of J8-Lipo-DT. The detailed physico-chemical properties of the liposomes and methodologies are described in Materials and Methods (and see Fig. 1).

**Figure 2:**
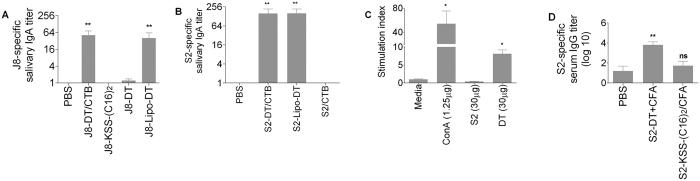
J8 and S2-specific antibody response and spleen cell proliferative response to antigens for individual BALB/c mice (n = 5/group). Mean antibody titer + SEM are shown. (**A**) J8-specific salivary IgA antibody response for BALB/c mice (n = 5/group). Mean antibody titers are represented as a bar + SEM. (**B**) S2-specific salivary IgA titer. (**C**) Average stimulation index (SI) of spleen cell proliferation for BALB/c mice immunized with 30 μg of S2 peptide and 30 μg of DT in complete Freund’s adjuvant. Proliferation of cells induced by S2 peptide and DT (30 μg) and concanavalin A (conA) was determined. The average counts per minute observed for control mice in the absence of antigen were 5303 cpm. (**D**) S2-specific serum IgG titer. Statistical analysis was performed using a nonparametric, unpaired Mann-Whitney U test to compare test groups to the PBS control group (ns, *p* > 0.05; *p < 0.05; **p < 0.01; ***p < 0.001).

We initially tested single peptide liposomes and asked whether J8 incorporated with the lipid bilayer of liposomes enclosing DT would induce a mucosal IgA response. J8 was attached to the liposome by addition to the J8 sequence of a spacer (KSS) and a di-palmitic acid tail ([C16]_2_). We observed that J8-KSS-(C16)_2_-Lipo-DT (abbreviated as J8-Lipo-DT) and J8 chemically conjugated to DT and mixed with CTB (J8-DT/CTB), but not lipidated J8 alone [J8-KSS-(C16)_2_] nor J8 chemically conjugated to DT without CTB (J8-DT) induced mucosal IgA responses following intra-nasal administration and two boosts (Fig. 2A). Although not relevant to streptococcal immunity, we also measured the fecal IgA responses and observed significant IgA responses in J8-Lipo-DT-vaccinated mice but not mice immunized with J8-KSS-(C16)_2_ (data not shown). These results show that the liposome construct was immunogenic, but that both lipidated J8 (without liposome with enclosed DT) and J8-DT (without liposomes) were not immunogenic and suggested that the liposome membrane was essential for induction of the IgA response. The results did not exclude a role for the lipid tail of the peptide; rather they show that it alone was not effective.

We then tested a different single peptide liposome, S2-KSS-(C16)_2_-Lipo-DT (abbreviated as S2-Lipo-DT). S2 is the 20-mer B-cell epitope from SpyCEP. We observed that the salivary S2-specific IgA response following intranasal vaccination was also significant and equivalent to that induced by S2 that had been chemically conjugated to DT (S2-DT) and administered with CTB (Fig. 2B). We were interested to know whether the immunogenicity of the construct was related to inherent immunogenicity of the S2 peptide. We observed that mice immunized intra-nasally with S2 (no lipid tail) mixed with CTB did not induce S2-specific IgA (Fig. 2B). The lack of response to S2/CTB, together with the positive response to S2-Lipo-DT, suggested that S2 alone was incapable of stimulating T-cells necessary to provide B-cell help. This was confirmed by immunizing mice with S2 and showing that spleen cells from immunized mice did not proliferate *in vitro* to S2 (Fig. 2C); and further confirmed by immunizing mice parenterally with S2-KSS-(C16)_2_ emulsified in complete Freund’s adjuvant (CFA) and showing a complete lack of an antibody response to S2. (Fig. 2D). These data with J8 and S2 collectively showed that a liposome encapsulating DT was essential for the IgA response and that the surface peptide is not required to activate T-cells.

The size, shape and surface charge of particulate vaccines can influence their immunogenicity and NLRP3 inflammasome activation is a feature of charged particulate vaccine adjuvants including liposomes^26^,^27^,^28^. We tested different sizes of J8-Lipo-DT and observed that the self-assembling microparticulate sized J8-Lipo-DT (1756 nm) and the 823 nm sized J8-Lipo-DT stimulated the highest IgA response (Supplemental Fig. 2). For all further experiments we used the larger sized liposomes.

We were interested to test whether a lyophilized vaccine would remain immunogenic post re-hydration. This would greatly facilitate development of the vaccine and negate any concerns that might arise due to instability of the liquid form of the vaccine^29^. We freeze-dried the vaccine, stored it at 4 °C for 1, 5 and 7 weeks, and re-suspended it in PBS prior to immunization. We observed that immunogenicity was completely maintained providing trehalose (a lyoprotectant) was added to the vaccine prior to freeze-drying (Supplemental Fig. 3A,B, which shows the data for 7 weeks post freeze-drying).

### Assessing protection from streptococcal infection

**Figure 3:**
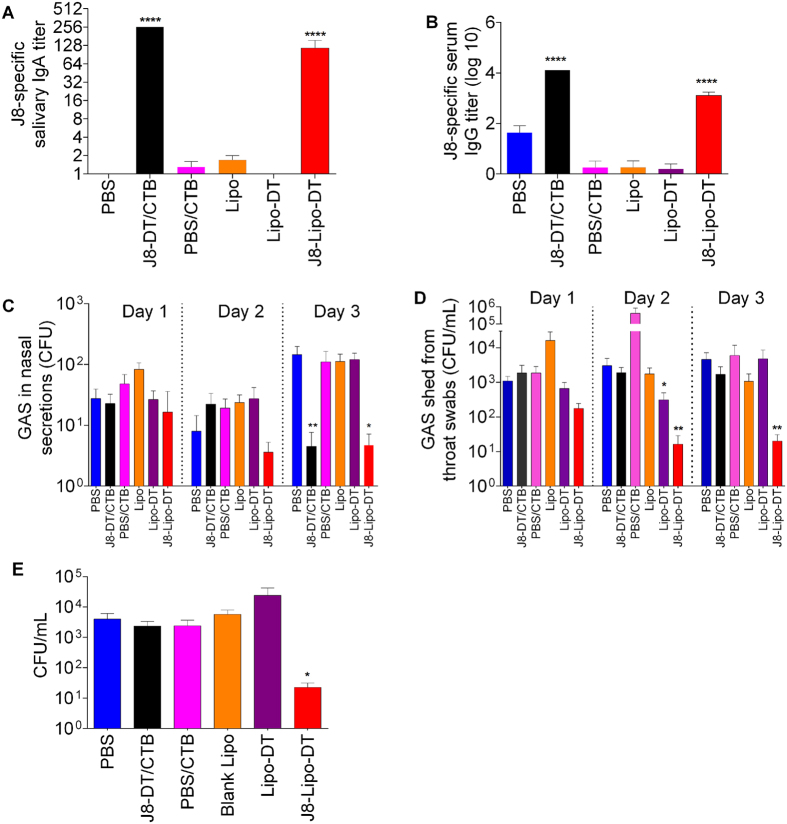
J8-specific antibody response and bacterial burden after intranasal challenge with M1 GAS strain in BALB/c mice (n = 10/group). Mean antibody titers are represented as a bar + SEM. Bacterial burden results are represented as the mean CFU + SEM for 10 mice/group on days 1–3 for throat swabs and nasal shedding, and day 3 for NALT. (**A**) Salivary IgA titer. (**B**) Serum IgG titer. (**C**) Nasal shedding. (**D**) Throat swabs. (**E**) Colonization of NALT. Statistical analysis was performed using a nonparametric, unpaired Mann-Whitney U test to compare test groups to the PBS control group (ns, *p* > 0.05; *p < 0.05; **p < 0.01; ***p < 0.001).

To assess protection, mice were immunized with J8-Lipo-DT while control groups were immunized with liposomes alone, liposomes encapsulating DT (Lipo-DT), J8-DT/CTB, DT/CTB or PBS. Mice were given a primary immunization followed by two booster immunizations (all delivered intra-nasally) and then challenged intra-nasally with a serotype M1 pharyngeal isolate (not a CovR/S mutant) obtained from a patient with scarlet fever^15^. Prior to challenge, we observed that J8-Lipo-DT and J8-DT/CTB induced comparable J8-specific salivary IgA and serum IgG (Fig. 3A,B). Post-challenge with GAS, the bacterial load in nasal discharge was significantly lower in mice immunized with either J8-Lipo-DT or J8-DT/CTB (Day 3, Fig. 3C). J8-Lipo-DT-immunized mice also showed significant protection against infection on the pharyngeal surface (throat swabs) and NALT (Fig. 3D,E). Surprisingly, however, J8-DT/CTB-immunized mice were not protected from colonization of the throat or NALT.

### Inflammatory responses following liposome vaccination

**Figure 4:**
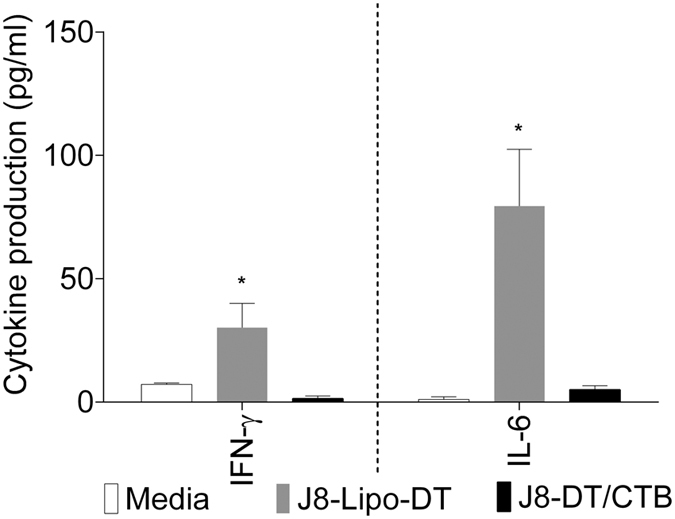
Antigen-specific secreted chemokines and cytokines in J8-Lipo-DT and J8-DT/CTB immunized mice. Splenocytes were plated out and the following stimuli were added as indicated: J8 (10 μg/ml) or media alone. 72 hours post-stimulation, supernatants were isolated and levels of secreted chemokines or cytokines were assayed using a cytometric bead array (see Materials and Methods). Statistical analysis was performed using a Student’s t test (ns, *p* > 0.05; *p < 0.05; **p < 0.01).

To explore potential mechanisms of protection, we measured the pro-inflammatory cytokine responses (IFN-γ, IL-1, IL-6, IL-12p70, MCP-1, and TNF-α) of spleen cells from immunized mice following *in vitro* stimulation with J8 (Fig. 4). J8 was able to recall IFN-γ and IL-6 responses from spleen cells from J8-Lipo-DT immunized mice, but not from mice immunized with J8-DT/CTB. The other cytokines tested were not detected. IL-6 is known to be a signal for neutrophil production^30^ and neutrophils are essential for IgA-mediated opsonization of GAS^31^. IL-6 can also augment IgA secretion^32^ and IFN-γ promotes IL-6 production^33^. We thus immunized IL6−/− and control mice with J8-Lipo-DT and observed that although both groups produced similar levels of J8-specific IgA (preliminary data), the IL6−/− mice had significantly higher total streptococcal tissue bioburden (pharynx, NALT and lungs) post-challenge in comparison to wildtype mice (unpublished observation). The data suggest the importance of inflammatory cellular responses in J8-Lipo-DT-mediated immunity and provided a likely explanation for the difference in protection against GAS in the tissues following J8-Lipo-DT and J8-DT/CTB vaccination, both of which induced similar levels of IgA.

### Immunogenicity of multi-epitope vaccine

**Figure 5:**
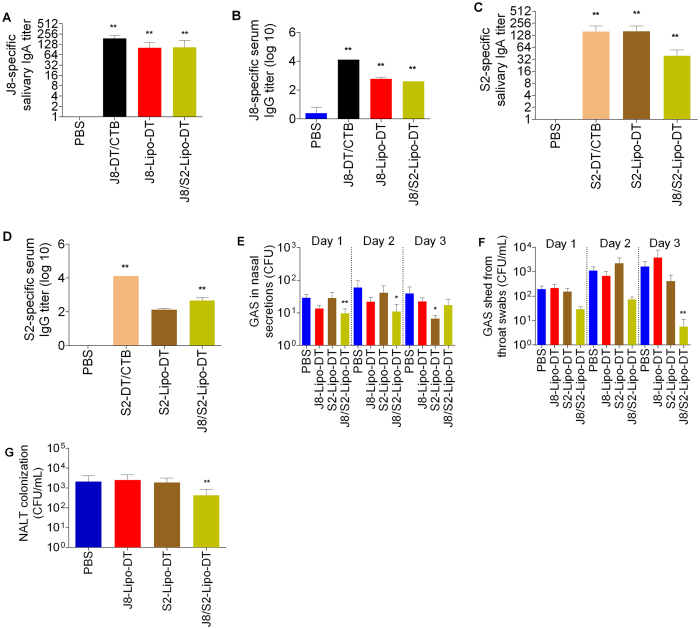
J8 and S2-specific antibody response and bacterial burden after intranasal challenge with 5448AP GAS strain in BALB/c mice (n = 10/group). Mean antibody titer + SEM are shown. (**A**) J8-specific salivary IgA titer. (**B**) J8-specific serum IgG titer. (**C**) S2-specific salivary IgA titer. (**D**) S2- serum IgG titer. (**E**) Nasal shedding. (**F**) Throat swabs. (**G**) Colonization of NALT. Statistical analysis was performed using a nonparametric, unpaired Mann-Whitney U test to compare test groups to the PBS control group (ns, *p* > 0.05; *p < 0.05; **p < 0.01; ***p < 0.001).

CovR/S mutant GAS have significantly enhanced virulence due to up-regulation of various virulence factors, including SpyCEP. Parenteral immunization with J8-DT/alum does not protect against CovR/S mutant GAS skin infection but co-immunization with J8-DT and S2-DT co-administered with alum does protect^12^. We asked whether liposomes expressing both J8 and S2 would induce IgA to both peptides and mediate mucosal protection. We constructed J8/S2-Lipo-DT (See Fig. 1) and compared its immunogenicity to the individual peptide liposomes (J8-Lipo-DT; S2-Lipo-DT [see Fig. 1]). We observed that the J8/S2- Lipo-DT construct induced comparable immune responses to both J8 and S2 as individual peptide constructs (Fig. 5A–D). However, following a challenge infection with GAS strain 5448AP (a CovR/S mutant), only mice immunized with the multi-epitope construct were protected in the throat and NALT (Fig. 5F,G). When measuring colonies in nasal secretions we observed that the number of organisms was small in the control mice but on two of the three days measured there was a significant reduction in colonies from mice that had received J8/S2-Lipo-DT immunization (Fig. 5E).

## Discussion

We have produced a mucosally active subunit liposomal vaccine formulation that can co-present more than one epitope, can activate both humoral and cellular protective immune responses to a major bacterial pathogen, and can be stored as a powder. The immunostimulatory properties of liposomes were exploited to encapsulate proteins and display lipidated molecules on their surface. Vaccine design readily facilitated the inclusion of additional B-cell epitopes in the lipid bi-layer thus enabling modular construction of a multi-epitope vaccine. Mucosal immunity induced by the liposome vaccine was superior to that induced by a peptide-protein conjugate administered with CTB, which correlated with the stimulation of an inflammatory response by spleen cells from liposome-vaccinated mice. The data showed that IL-6 was induced by the liposome vaccine and suggested that it was a critical cytokine as mice immunized with J8-DT/CTB produced very similar levels of IgA but had much lower levels of IL-6 post-antigen stimulation *in vitro* and were not protected. Furthermore, vaccinated IL-6−/− mice were protected significantly less well than vaccinated control mice in preliminary data.

Mucosal immunization, as a means of eliciting protective immunity against infectious diseases, has attracted much interest. The vast majority of infections occur at, or begin from, mucosal surfaces. Therefore, a vaccine that can induce a mucosal protective immune response is highly desirable. However, in practice it has proven difficult to stimulate strong mucosal IgA responses by vaccination and progress in development of mucosal vaccines using subunit peptide antigens has been disappointing. Addition of toxic adjuvants (including enterotoxins such as CTB and the Labile Toxin of *E. coli* [LT]) and conjugation of subunit antigens to protein carriers to provide T-cell help are essential.

Previous liposomal research involved vaccine construction using cationic liposomes with peptide antigens and the use of linked ‘universal’ T-cell epitope peptides to induce T-cell help^34^,^35^. These are useful approaches but lack the utility of the Peptide-Lipo-DT approach where additional B-cell peptide epitopes can be readily displayed on the liposomal surface and do not require chemical conjugation to a T-cell peptide epitope. It is also unknown whether or not the short universal peptide T-epitopes will induce a T-helper cell response in the majority of humans, whereas DT is a well-recognized, widely immunogenic carrier protein/vaccine. Liposomes reliant on universal peptide epitopes have not been tested for their ability to induce protective immune responses to GAS. Other liposomes without adjuvants were previously reported to deliver encapsulated peptides to induce a cellular immune response, but such liposomes do not induce an IgA nor an IgG response^36^,^37^. No other reported studies with liposomes displaying peptides of multiple specificities on the surface of a single construct have been reported, to our knowledge. It is possible that the lipid tails on J8 and S2 provided some adjuvant activity contributing to the immunogenicity; however, the lipidated peptide on its own was not immunogenic demonstrating the need for the liposome membrane. Lipopeptides do engage TLR2^19^ and this, together with liposomal activation of the NLRP3 inflammasome^28^ may be critical to innate immune stimulation. The topographical position of liposome-associated antigens affects antigen processing and presentation to B and helper T-cells^38^. It has been demonstrated that antigens exposed on the liposome surface are preferentially processed and presented to B-cells whilst liposome-encapsulated antigens are more effectively processed and presented to T-cells by antigen presenting cells^39^. As such, Peptide-Lipo-DT represents a rational subunit vaccine design, ensuring surface B-cell epitopes are exposed to and bind the Ig receptor of a B-cell whilst encapsulation of DT allows effective delivery, processing and presentation to T-cells.

The liposome vaccine induces an inflammatory response that may be critical to enhanced protection. We demonstrated clearance of GAS in the URT tissue (throat and NALT) following challenge of liposome-vaccinated mice. There was significantly less protection in the J8-DT/CTB-immunized mice (Fig. 3) although there was a reduction of GAS in nasal secretions. This may reflect a need for the cytokine inflammatory response, in particular IL-6 (in response to GAS antigen stimulation), to clear the infection from the tissue^40^,^41^, whereas GAS in nasal secretions may be neutralized by IgA. IL-6 plays a critical role in bacterial immunity by stimulating bone marrow production of neutrophils^30^. Immunity to GAS depends critically on neutrophil migration to the site of infection^42^, and human IgA antibodies specific to the conserved region of the M-protein require neutrophils to kill GAS^31^. Therefore, mechanisms that are fundamental to conferring immunity in humans to GAS infection may be induced using the liposome platform. As such, the data presented here, argue that a J8/S2-Lipo-DT vaccine will be effective in controlling human GAS infections of the URT.

The use of liposomes to induce mucosal immunity has received attention from the perspective of safety. Concerns were raised after a nasal influenza vaccine, containing Labile Toxin of *E. coli* (LT) as an adjuvant, was associated with a higher incidence of Bell’s palsy (facial nerve paralysis)^43^. It was suggested that this was due to the neurotoxic effects of neuronal ganglioside-binding LT and possible reactivation of latent viral infections^44^. In support of this, two individuals in a later study also developed a transient Bell’s palsy after administration of liposomes containing LT^45^. It seems most unlikely that intranasal immunization *per se* would have deleterious safety concerns given the lack of any reports of Bell’s palsy in individuals receiving other intranasal vaccines not containing LT and the fact that the nose receives more infectious insults than any other tissue of the body.

The vaccine approach described here could be used to induce immune responses against multiple antigens incorporating multiple peptides from a single pathogen or from various URT pathogens. Here we showed the utility of this multi-epitope approach by incorporating target peptides from two independent virulence factors of GAS.

In conclusion, the findings reported here represent an important step toward overcoming many current obstacles in the development of vaccines to prevent infection at mucosal sites. We provide mechanistic insights into how a novel liposomal vaccine induces the desired humoral and cellular mucosal immune responses to collectively combat a major mucosal pathogen. We further demonstrate its potential for the development of vaccines against other pathogenic mucosal organisms that are sensitive to a mucosal IgA response and/or a cellular response.

## Materials and Methods

### Ethics statement

All animal protocols used were approved by the Griffith University Animal Ethics Committee, GU Ref No: GLY/09/14/AEC. This study was carried out in accordance with the National Health and Medical Research Council (NHMRC) of Australia guidelines for the generation, breeding, care and use of genetically modified and cloned animals for scientific purposes (2007). Methods were chosen to minimize pain and distress to the mice and animals were observed daily by trained animal care staff. Mice were terminated using a CO_2_ inhalation chamber.

### J8 and S2-Lipo-DT formulation

To promote noncovalent complexing of J8 or S2 to liposome bilayer, a hydrophobic anchor consisting of two palmitic acids (C16) was added to the epsilon and primary amine group of the lysine in a tripeptide spacer (consisting of Lys Ser Ser) present in J8 or S2 amino-terminus (S2-KSS-(C16)_2_ or J8-KSS-(C16)_2_). Net charge of peptides including the Lys Ser Ser spacer was done using PepCalc (http://pepcalc.com/ppc.php) and reported as +3 for J8-KSS and −6 for S2-KSS. These constructs were manufactured by Chinapeptides Co., Ltd. (Shanghai, China). The expected molecular weight of the construct (J8-KSS-(C16)_2_: MW 4061.97 g/mol and S2-KSS-(C16)_2_: MW 3314.75 g/mol) was confirmed by ESI-MS, and the product was obtained at greater than 95% purity (by analytical RP-HPLC area under the curve analysis). Liposomes were prepared using the thin film hydration method^46^. Neutral lipids from Avanti Polar Lipids, Inc. (Alabaster, United States) were used at a molar ratio of 7 dipalmitoyl-sn-glycero-3-phosphocholine (DPPC): 2 Cholesterol (CHOL): 1 L-α-phosphatidylglycerol (PG). Lipids in chloroform (CHCl_3_) solution were coated onto round-bottom flasks using a rotary evaporator along with predetermined amounts of S2-KSS-(C16)_2_ or J8-KSS-(C16)_2_. The volumes used were 0.7 ml of DPPC (10 mg/ml) in CHCl_3_, 0.2 ml of CHOL (5 mg/ml) in CHCl_3_, and 0.1 ml of PG (10 mg/ml) in CHCl_3_. The lipid thin film was then hydrated and dispersed in 1 ml of phosphate buffered saline (PBS) containing a predetermined amount of DT by vigorous mixing at room temperature. The resultant liposomal suspension was centrifuged at 16,162 *g* for 10 min, the supernatant removed, and the liposome pellet resuspended in an appropriate volume of PBS to be administered in mice. For different sized J8-Lipo-DT, prior to centrifugation, extrusion with a 0.1, 0.4 or 1 μm polycarbonate membrane was undertaken. To determine DT encapsulation efficiency, the supernatant was collected and the amount of unentrapped DT was determined using a NanoDrop 2000 UV-Vis Spectrophotometer (Thermo Scientific, Massachusetts, United States). Subtraction of supernatant DT concentration from starting DT concentration in PBS used for rehydration of lipids to produce liposomes allowed quantification of encapsulation efficiency. The average particle size (nm) of a liposome was measured at 25 °C using a Nanosizer (Zetasizer Nano Series ZS, Malvern Instruments, United Kingdom) with disposable capillary cuvettes. Size was analyzed using a non-invasive backscatter system and measurements taken with a 173° scattering angle. Correlation times were based on 10 seconds per run and at least five consecutive runs were made per measurement. The results are the average of triplicate independent measurements analysed using Dispersion Technology Software (Malvern Instruments, United Kingdom). Homogenous size distribution as determined by a low polydispersity index (PDI) of 0.238 was shown for J8-Lipo-DT with an average size of 1756 nm (standard deviation of 100.3 nm). The PDI is an indication of how narrow the sample size distribution is and values greater than 0.7 indicate samples with a broad size distribution. For a molecule to molecule ratio of peptides to DT, amino acid analysis was reported by an independent third party contractor (The Australian Proteome Analysis Facility, Australia). Fluorescent liposomes for encapsulation of DT consisted of J8-KSS-(C16)_2_ attached to the fluorophore fluorescein isothiocyanate manufactured by Chinapeptides Co., Ltd. (Shanghai, China). The expected molecular weight of the construct (MW 4692.69 g/mol) was confirmed by ESI-MS, and the product was obtained at greater than 95% purity (by analytical RP-HPLC area under the curve analysis). To determine percentage of DT associated with liposomes, DT was labelled with CF™ 405S succinimidyl ester (Sigma Aldrich, St. Louis, United States) according to the manufacturer’s recommendations. Liposomes were labeled with the phospholipid stain DiI (1,1′-dioctadecyl-3,3,3′,3′-tetramethylindocarbocyanine perchlorate) (Vybrant^®^, Life Technologies, CA, USA) by addition of 1 μl of DiI to J8-Lipo-DT in PBS, and incubated at 4 °C for 30 min, then subjected to two rounds of centrifugation at 16,162 *g* for 10 min. The samples were analyzed in a CyAn ADP analyzer (Beckman Coulter).

### Intra-nasal immunization of mice

BALB/c, C57BL/6 mice (Animal Resources Centre, Western Australia, Australia) and C57BL/6 IL-6 −/− mice (gifted by Prof. Geoffrey Hill) were anesthetized by use of a mixture of xylazine and ketamine (1:1:10 mixture of xylazine: ketamine: H_2_O). Mice were administered formulations of J8-Lipo-DT, S2-Lipo-DT or J8/S2-Lipo-DT alone in a total volume of 20 μL PBS (10 μL/nare) containing 30 μg of peptide (J8-KSS-(C16)_2_, S2-KSS-(C16)_2_) and 17 μg of DT whilst control mice were administered 20 μL of PBS (10 μL/nare). Positive control mice received 30 μg of J8-DT or S2-DT, co-administered with 10 μg of CTB (Sigma Aldrich, St. Louis, United States) in a total volume of 20 μL PBS. The mice received 2 booster immunizations 21 days apart in the same method as the primary immunization. Other control groups received equivalent amounts of J8, DT or liposome alone as described above.

### Intra-muscular immunization of mice

BALB/c mice received primary immunization and two boosts with J8-DT/alum on day 0, 21 and 28 (according to the primary immunization schedule). All immunizations were given intra-muscularly and a total of 30 μg of J8-DT was administered. The J8-DT/alum and PBS/alum formulation were prepared fresh before each immunisation. J8-DT (10 mg/ml stock) and PBS were adsorbed on to alhydrogel (alum; Brenntag Biosector, Denmark) in a 1:1 ratio. The J8-DT/alum and PBS/alum preparations were left to rotate for an hour at room temperature (RT). The J8-DT/alum and PBS/alum vaccine formulations were administered (30 μg antigen in a 50 μl volume). Briefly, mice were anesthetized by use of a mixture of xylazine and ketamine (1:1:10 mixture of xylazine: ketamine: H_2_O). The haunch (central in the ventral face of the left leg muscle) of the mice was then sterilised with 80% ethanol (EtOH) and the muscle was then pinched up to form a bulge. With a needle positioned vertically, in to the bulge of muscle, 50 μl of antigen preparation was injected. Boosts were applied in the opposite limb to the previous immunisation.

### Serum, saliva and fecal sample collection

Serum was collected on days 20, 40, and 60 after primary immunization to determine the level of J8-specific systemic antibodies. Blood was collected from mice via the tail artery and allowed to clot for at least 30 min at 37 °C. Serum was collected after centrifugation for 10 min at 1000 *g* and stored at −20 °C.

Mice were administered 50 μL of a 0.1% solution of pilocarpine to induce salivation. Saliva was then collected in eppendorf tubes containing 2 μL of 50 mmol/L phenylmethylsulfonyl fluoride (PMSF) protease inhibitor (Sigma Aldrich). Particulate matter was separated by centrifugation for 10 min at 13,000 *g* and samples were stored at −80 °C.

Six to 10 freshly voided fecal pellets were collected from individual mice, frozen and then lyophilized. The dry weight of fecal solids was determined before they were resuspended by vortexing in 5% nonfat dry milk, 50 mmol/L EDTA (Sigma Aldrich), 0.1 mg/mL soyabean trypsin inhibitor (Sigma Aldrich), and 2 mmol/L PMSF (20 μL/mg of dry weight). Solid matter was separated by centrifugation for 10 min at 15,000 *g*. The supernatants were stored at −80 °C.

### Determination of antibody titers by enzyme-linked immunosorbent assay (ELISA)

ELISA was used to measure J8-specific serum IgG and mucosal IgA as described elsewhere^47^. J8 and S2 peptides were diluted to 0.5 mg/ml in carbonate coating buffer, pH 9.6, and coated onto polycarbonate plates in a volume of 100 μl/well overnight at 4 °C. Unbound peptide was removed and the wells blocked with 150 μl of 5% skim milk PBS-Tween 20 for 2 h at 37 °C. The plates were then washed 3 times with PBS-Tween 20 buffer. Samples were serially diluted down the plate in 0.5% skim milk PBS-Tween 20 buffer, starting at an initial dilution of 1:100 to a final dilution of 1:12,800 for sera and 1:2 to 1:256 for saliva and fecal samples. Each sample was diluted to a final volume of 100 μl and incubated for 1.5 h at 37 °C. The plates were washed 5 times and peroxidase-conjugated goat anti-mouse IgG or IgA (Invivogen, San Diego, United States) were added at a dilution of 1:3000 or 1:1000 respectively in 0.5% skim milk PBS-Tween 20 for 1.5 h at 37 °C. After washing, 100 μl of OPD substrate (Sigma Aldrich) was added according to the manufacturer’s instructions and incubated at room temperature for 30 min in the dark. The absorbance was measured at 450 nm in a Victor^3^ 1420 multilabel counter (Perkin Elmer Life and Analytical Sciences, Shelton, United States). The titer was described as the lowest dilution that gave an absorbance of >3 standard deviations (SD) above the mean absorbance of negative control wells (containing normal mouse serum immunized with PBS). Statistical significance (*p* < 0.05) was determined using an unpaired Mann-Whitney U test to compare test groups to the PBS control group (p < 0.05 was considered significant) using GraphPad Prism 5 software (GraphPad, California, United States).

### Procedure for GAS challenge

Immunized and control mice were challenged intra-nasally with a predetermined dose of the GAS strain M1 on day 63 after primary immunization. The GAS strain M1 had been serially passaged in mouse spleen to enhance virulence, and made streptomycin-resistant to enable GAS to be distinguished in throat swabs from normal murine bacterial flora^48^. The GAS strains 5448AP was obtained from the Walker lab. To determine GAS colonization, throat swabs were obtained from mice on days 1–3 after challenge. The throat swabs were streaked out on Columbia base agar plates containing 2% defibrinated horse blood and incubated overnight at 37 °C. Bacterial burden in nasal shedding was determined by pressing the nares of each mouse onto the surface of Columbia blood agar (CBA) plates ten times (triplicate CBA plates/mouse/day) and exhaled particles were streaked out^49^. On day 3 mice were culled, organ samples were homogenized in PBS and samples were plated in triplicate using the pour plate method. For nasal shedding and throat swabs, results are represented as the mean colony forming units (CFU) + standard errors of the means (SEM) for 10–15 mice/group on days 1, 2 and 3. For organ samples, results are represented as the mean CFU + SEM for 10 mice/group on day 3. Differences were analysed with GraphPad Prism 5 using a nonparametric, unpaired Mann-Whitney U test to compare test groups to the PBS control group (*p* < 0.05 was considered significant).

### *In vitro* stimulation of splenocytes with antigen

The cytometric bead array (CBA) assay and flow cytometry analysis were used to quantify the pro-inflammatory response produced by splenocytes after stimulation with the J8 peptide. Briefly, single-cell suspensions of spleens from J8-Lipo-DT-immunized mice free of erythrocytes were prepared in RPMI 1640 media. Splenocytes (4 × 10^5^) in a total volume of 0.1 ml were plated out and the following stimuli were added as indicated: LPS (Sigma Aldrich) at 2 μg/ml, J8 (10 μg/ml), or RPMI 1640 media alone for 72 h. Supernatants were isolated after 72 h and stored at −80 °C for CBA flow cytometric analysis.

### Quantification of secreted chemokines and cytokines by CBA

Levels of accumulated inflammatory cytokines were quantified according to the manufacturer’s instructions. For the Mouse Inflammation Kit CBA, volumes of samples and standards were scaled down to 10 μl, and 2 μl of each capture bead was used. The samples were analyzed in a CyAn ADP analyzer (Beckman Coulter) and data analysed with FCAP array (v1.01 for Windows) software (Becton Dickinson). The data are reported as means + standard errors of the means (SEM), and differences were analysed with GraphPad Prism 5 software using Student’s t test. *P* values under 0.05 were considered significant.

### Spleen cell proliferation assay

Briefly, S2 peptide and DT was administered subcutaneously at the tail base, with each mouse receiving 30 μg of peptide and DT emulsified in CFA. Ten days later, spleen cells were plated in quadruplicate at 4 × 10^5^ cells/well in RPMI 1640 complete medium containing 5 × 10^−5^ M β–mercaptoethanol and antigen at various concentrations. The plates were incubated for 4 days in a humidified 5% CO_2_/air environment at 37 °C. During the last 18 h of culture, cells were pulsed with 0.25 μCi of ^3^[H]-Thymidine (PerkinElmer)/well and harvested onto fiberglass mats; ^3^H incorporation was measured on a PerkinElmer MicroBeta2 β counter. Stimulation index (SI) was defined as counts per minute in the presence of antigen/counts per minute in the absence of antigen.

### Freeze-drying of liposomes

Freshly prepared liposomes were freeze-dried in 0.4 ml of Milli-Q water (Millipore, 18.2 MΩ cm at 25 °C) in glass vials with 10% Trehalose (w/w). The vials were frozen in dry ice, dissolved in acetone for 10 min and placed on the plate of a freeze-dryer with a temperature of −40 °C. At the end of the freeze-drying process the glass vials were closed with plastic cap and stored at 4 °C.

### Statistics

Statistical analysis was performed with GraphPad Prism 5 software using a nonparametric, unpaired Mann-Whitney U test (one-tailed) to compare test groups to the PBS control group (ns, *p* > 0.05; *p < 0.05; **p < 0.01; ***p < 0.001).

## Additional Information

**How to cite this article:** Zaman, M. *et al*. Novel platform technology for modular mucosal vaccine that protects against streptococcus. *Sci. Rep.* **6**, 39274; doi: 10.1038/srep39274 (2016).

**Publisher’s note:** Springer Nature remains neutral with regard to jurisdictional claims in published maps and institutional affiliations.

## Supplementary Material

Supplementary Materials

## References

[b1] Gonzales R., Steiner J. F. & Sande M. A. Antibiotic prescribing for adults with colds, upper respiratory tract infections, and bronchitis by ambulatory care physicians. Jama 278, 901–904 (1997). 9302241

[b2] Global, regional, and national age-sex specific all-cause and cause-specific mortality for 240 causes of death, 1990–2013: a systematic analysis for the Global Burden of Disease Study 2013. *Lancet* **385**, 117–171, doi: 10.1016/s0140-6736(14)61682-2 (2015). PMC4340604 25530442

[b3] Steer A. C., Dale J. B. & Carapetis J. R. Progress toward a global group a streptococcal vaccine. Pediatr Infect Dis J 32, 180–182, doi: 10.1097/INF.0b013e318281da11 (2013). 23328823

[b4] Metzgar D. & Zampolli A. The M protein of group A Streptococcus is a key virulence factor and a clinically relevant strain identification marker. Virulence 2, 402–412, doi: 10.4161/viru.2.5.16342 (2011). 21852752

[b5] Dale J. B. . Potential coverage of a multivalent M protein-based group A streptococcal vaccine. Vaccine 31, 1576–1581, doi: 10.1016/j.vaccine.2013.01.019 (2013). 23375817 PMC3593940

[b6] Batzloff M. R. . Protection against group A streptococcus by immunization with J8-diphtheria toxoid: contribution of J8- and diphtheria toxoid-specific antibodies to protection. The Journal of infectious diseases 187, 1598–1608, doi: 10.1086/374800 (2003). 12721940

[b7] Guilherme L., Ferreira F. M., Kohler K. F., Postol E. & Kalil J. A vaccine against Streptococcus pyogenes: the potential to prevent rheumatic fever and rheumatic heart disease. Am J Cardiovasc Drugs 13, 1–4, doi: 10.1007/s40256-013-0005-8 (2013). 23355360

[b8] Turner C. E., Kurupati P., Wiles S., Edwards R. J. & Sriskandan S. Impact of immunization against SpyCEP during invasive disease with two streptococcal species: Streptococcus pyogenes and Streptococcus equi. Vaccine 27, 4923–4929, doi: 10.1016/j.vaccine.2009.06.042 (2009). 19563892 PMC2759039

[b9] Shet A., Kaplan E. L., Johnson D. R. & Cleary P. P. Immune response to group A streptococcal C5a peptidase in children: implications for vaccine development. J Infect Dis 188, 809–817, doi: 10.1086/377700 (2003). 12964111

[b10] Auzanneau F. I., Borrelli S. & Pinto B. M. Synthesis and immunological activity of an oligosaccharide-conjugate as a vaccine candidate against Group A Streptococcus. Bioorg Med Chem Lett 23, 6038–6042, doi: 10.1016/j.bmcl.2013.09.042 (2013). 24103300

[b11] Hayman W. A. . Mapping the minimal murine T cell and B cell epitopes within a peptide vaccine candidate from the conserved region of the M protein of group A streptococcus. Int Immunol 9, 1723–1733 (1997). 9418133 10.1093/intimm/9.11.1723

[b12] Pandey M. & Mortensen R. Combinatorial Synthetic Peptide Vaccine Strategy Protects against Hypervirulent CovR/S Mutant Streptococci. 196, 3364–3374, doi: 10.4049/jimmunol.1501994 (2016). 26969753

[b13] Pandey M. . A synthetic M protein peptide synergizes with a CXC chemokine protease to induce vaccine-mediated protection against virulent streptococcal pyoderma and bacteremia. J Immunol 194, 5915–5925, doi: 10.4049/jimmunol.1500157 (2015). 25980008

[b14] Bessen D. & Fischetti V. A. Passive acquired mucosal immunity to group A streptococci by secretory immunoglobulin A. J Exp Med 167, 1945–1950 (1988). 3290383 10.1084/jem.167.6.1945 PMC2189674

[b15] Batzloff M. R. . Toward the development of an antidisease, transmission-blocking intranasal vaccine for group a streptococcus. J Infect Dis 192, 1450–1455, doi: 10.1086/466528 (2005). 16170764

[b16] Lee S. & Nguyen M. T. Recent advances of vaccine adjuvants for infectious diseases. Immune Netw 15, 51–57, doi: 10.4110/in.2015.15.2.51 (2015). 25922593 PMC4411509

[b17] Moschos S. A., Bramwell V. W., Somavarapu S. & Alpar H. O. Adjuvant synergy: the effects of nasal coadministration of adjuvants. Immunol Cell Biol 82, 628–637, doi: 10.1111/j.0818-9641.2004.01280.x (2004). 15550121

[b18] Hussein W. M., Liu T. Y., Skwarczynski M. & Toth I. Toll-like receptor agonists: a patent review (2011 - 2013). Expert Opin Ther Pat 24, 453–470, doi: 10.1517/13543776.2014.880691 (2014). 24456079

[b19] Zaman M. & Toth I. Immunostimulation by synthetic lipopeptide-based vaccine candidates: structure-activity relationships. Front Immunol 4, 318, doi: 10.3389/fimmu.2013.00318 (2013). 24130558 PMC3793171

[b20] Tandrup Schmidt S., Foged C., Korsholm K. S., Rades T. & Christensen D. Liposome-Based Adjuvants for Subunit Vaccines: Formulation Strategies for Subunit Antigens and Immunostimulators. Pharmaceutics 8, doi: 10.3390/pharmaceutics8010007 (2016). PMC4810083 26978390

[b21] Watson D. S., Endsley A. N. & Huang L. Design considerations for liposomal vaccines: influence of formulation parameters on antibody and cell-mediated immune responses to liposome associated antigens. Vaccine 30, 2256–2272, doi: 10.1016/j.vaccine.2012.01.070 (2012). 22306376 PMC3296885

[b22] Giddam A. K., Zaman M., Skwarczynski M. & Toth I. Liposome-based delivery system for vaccine candidates: constructing an effective formulation. Nanomedicine (Lond) 7, 1877–1893, doi: 10.2217/nnm.12.157 (2012). 23249332

[b23] Yuseff M. I., Pierobon P., Reversat A. & Lennon-Dumenil A. M. How B cells capture, process and present antigens: a crucial role for cell polarity. Nat Rev Immunol 13, 475–486, doi: 10.1038/nri3469 (2013). 23797063

[b24] Ingale S., Wolfert M. A., Gaekwad J., Buskas T. & Boons G. J. Robust immune responses elicited by a fully synthetic three-component vaccine. Nat Chem Biol 3, 663–667, doi: 10.1038/nchembio.2007.25 (2007). 17767155 PMC2836923

[b25] Park H. S. & Cleary P. P. Active and passive intranasal immunizations with streptococcal surface protein C5a peptidase prevent infection of murine nasal mucosa-associated lymphoid tissue, a functional homologue of human tonsils. Infect Immun 73, 7878–7886, doi: 10.1128/iai.73.12.7878-7886.2005 (2005). 16299278 PMC1307028

[b26] Bachmann M. F. & Jennings G. T. Vaccine delivery: a matter of size, geometry, kinetics and molecular patterns. Nat Rev Immunol 10, 787–796, doi: 10.1038/nri2868 (2010). 20948547

[b27] Neumann S. . Activation of the NLRP3 inflammasome is not a feature of all particulate vaccine adjuvants. Immunol Cell Biol 92, 535–542, doi: 10.1038/icb.2014.21 (2014). 24687021

[b28] Zhong Z. . TRPM2 links oxidative stress to NLRP3 inflammasome activation. Nat Commun 4, 1611, doi: 10.1038/ncomms2608 (2013). 23511475 PMC3605705

[b29] Akbarzadeh A. . Liposome: classification, preparation, and applications. Nanoscale Res Lett 8, 102, doi: 10.1186/1556-276x-8-102 (2013). 23432972 PMC3599573

[b30] Walker F. . IL6/sIL6R complex contributes to emergency granulopoietic responses in G-CSF- and GM-CSF-deficient mice. Blood 111, 3978–3985, doi: 10.1182/blood-2007-10-119636 (2008). 18156493

[b31] Brandt E. R. . Functional analysis of IgA antibodies specific for a conserved epitope within the M protein of group A streptococci from Australian Aboriginal endemic communities. Int Immunol 11, 569–576 (1999). 10323210 10.1093/intimm/11.4.569

[b32] Beagley K. W. . Interleukins and IgA synthesis. Human and murine interleukin 6 induce high rate IgA secretion in IgA-committed B cells. J Exp Med 169, 2133–2148 (1989). 2786548 10.1084/jem.169.6.2133 PMC2189333

[b33] Yi A. K., Chace J. H., Cowdery J. S. & Krieg A. M. IFN-gamma promotes IL-6 and IgM secretion in response to CpG motifs in bacterial DNA and oligodeoxynucleotides. J Immunol 156, 558–564 (1996). 8543806

[b34] Ghaffar K. A. . Liposome-based Intranasal Delivery of Lipopeptide Vaccine Candidates Against Group A Streptococcus. Acta Biomater, doi: 10.1016/j.actbio.2016.04.012 (2016). 27063491

[b35] Zaman M. . Structure-activity relationship for the development of a self-adjuvanting mucosally active lipopeptide vaccine against Streptococcus pyogenes. J Med Chem 55, 8515–8523, doi: 10.1021/jm301074n (2012). 22974133

[b36] White W. I. . Antibody and cytotoxic T-lymphocyte responses to a single liposome-associated peptide antigen. Vaccine 13, 1111–1122 (1995). 7491819 10.1016/0264-410x(94)00058-u

[b37] Ninomiya A., Ogasawara K., Kajino K., Takada A. & Kida H. Intranasal administration of a synthetic peptide vaccine encapsulated in liposome together with an anti-CD40 antibody induces protective immunity against influenza A virus in mice. Vaccine 20, 3123–3129 (2002). 12163263 10.1016/s0264-410x(02)00261-x

[b38] Dal Monte P. & Szoka F. C. Jr. Effect of liposome encapsulation on antigen presentation *in vitro*. Comparison of presentation by peritoneal macrophages and B cell tumors. J Immunol 142, 1437–1443 (1989). 2465339

[b39] Harding C. V., Collins D. S., Slot J. W., Geuze H. J. & Unanue E. R. Liposome-encapsulated antigens are processed in lysosomes, recycled, and presented to T cells. Cell 64, 393–401 (1991). 1899049 10.1016/0092-8674(91)90647-h

[b40] Dileepan T. . Robust antigen specific th17 T cell response to group A Streptococcus is dependent on IL-6 and intranasal route of infection. PLoS Pathog 7, e1002252, doi: 10.1371/journal.ppat.1002252 (2011). 21966268 PMC3178561

[b41] Dileepan T. . Group A Streptococcus intranasal infection promotes CNS infiltration by streptococcal-specific Th17 cells. J Clin Invest 126, 303–317, doi: 10.1172/jci80792 (2016). 26657857 PMC4701547

[b42] Terao Y., Yamaguchi M., Hamada S. & Kawabata S. Multifunctional glyceraldehyde-3-phosphate dehydrogenase of Streptococcus pyogenes is essential for evasion from neutrophils. J Biol Chem 281, 14215–14223, doi: 10.1074/jbc.M513408200 (2006). 16565520

[b43] Mutsch M. . Use of the inactivated intranasal influenza vaccine and the risk of Bell’s palsy in Switzerland. N Engl J Med 350, 896–903, doi: 10.1056/NEJMoa030595 (2004). 14985487

[b44] Couch R. B. Nasal vaccination, *Escherichia coli* enterotoxin, and Bell’s palsy. N Engl J Med 350, 860–861, doi: 10.1056/NEJMp048006 (2004). 14985482

[b45] Lewis D. J. . Transient facial nerve paralysis (Bell’s palsy) following intranasal delivery of a genetically detoxified mutant of Escherichia coli heat labile toxin. PLoS One 4, e6999, doi: 10.1371/journal.pone.0006999 (2009). 19756141 PMC2737308

[b46] Szoka F. Jr. & Papahadjopoulos D. Comparative properties and methods of preparation of lipid vesicles (liposomes). Annu Rev Biophys Bioeng 9, 467–508, doi: 10.1146/annurev.bb.09.060180.002343 (1980). 6994593

[b47] Zaman M. . Group A Streptococcal vaccine candidate: contribution of epitope to size, antigen presenting cell interaction and immunogenicity. Nanomedicine (Lond) 9, 2613–2624, doi: 10.2217/nnm.14.190 (2014). 25529566

[b48] Olive C., Clair T., Yarwood P. & Good M. F. Protection of mice from group A streptococcal infection by intranasal immunisation with a peptide vaccine that contains a conserved M protein B cell epitope and lacks a T cell autoepitope. Vaccine 20, 2816–2825 (2002). 12034109 10.1016/s0264-410x(02)00205-0

[b49] Alam F. M., Turner C. E., Smith K., Wiles S. & Sriskandan S. Inactivation of the CovR/S virulence regulator impairs infection in an improved murine model of Streptococcus pyogenes naso-pharyngeal infection. PLoS One 8, e61655, doi: 10.1371/journal.pone.0061655 (2013). 23637876 PMC3636223

