# Supplementary material for: Novel platform technology for modular mucosal vaccine that protects against streptococcus
Source: Sci Rep. 2016 Dec 15;6:39274. doi: 10.1038/srep39274 (PMC5157026; doi:10.1038/srep39274)
Supplement: Supplementary Materials [file srep39274-s1.docx]

**Supplementary materials:**

**Novel platform technology for modular mucosal vaccine that protects against streptococcus**

**Authors:** Mehfuz Zaman^1,*,¶^, Victoria Ozberk^1,¶^, Emma L. Langshaw^1^, Virginia McPhun^1^, Jessica L. Powell^1^, Zachary. N. Phillips^1^, Mei Fong Ho^1^, Ainslie Calcutt^1^, Michael R. Batzloff^1^, Istvan Toth^2, 3,4^, Geoffrey R. Hill^5,6^, Manisha Pandey^1^, and Michael F. Good^1,*^

**Affiliations:**

^1^Institute for Glycomics, Griffith University, Gold Coast, QLD 4222, Australia.

^2^The University of Queensland, School of Chemistry and Molecular Biosciences, St Lucia, QLD 4072, Australia.

^3^The University of Queensland, School of Pharmacy, Woolloongabba, QLD 4102, Australia.

^4^Institute for Molecular Biosciences, The University of Queensland, St Lucia, QLD 4072, Australia.

^5^QIMR Berghofer Medical Research Institute, QIMR Berghofer Centre for Immunotherapy and Vaccine Development, Brisbane QLD 4029, Australia.

^6^Bone Marrow Transplant Unit, Royal Brisbane Hospital, Brisbane, QLD 4006, Australia.

**Supplementary figure legends**

**

**

**Fig. S1.** J8 specific antibody response after intra-muscular immunization and repeat challenge data for bacterial burden after intra-nasal challenge with M1 GAS strain in BALB/C mice (n=15/group). Mean antibody titer + SEM are shown. Bacterial burden results are represented as the mean CFU + SEM for 15 mice/group on days 1-3 for throat swabs, nasal shedding and 5 mice/group for NALT. **A)** J8-specific serum IgG titer. **B)** J8-specific salivary IgA titer. **C**) Nasal shedding of mice challenged with M1 GAS. **D)** Throat swabs from mice challenged with M1 GAS. **E)** Colonization of NALT from mice challenged with M1 GAS. **F**) Nasal shedding of mice after repeat challenge with M1 GAS. **G)** Throat swabs after repeat challenge with M1 GAS. **H)** Colonization of NALT after repeat challenge with M1 GAS. Statistical analysis was performed using a nonparametric, unpaired Mann-Whitney U test to compare test groups to the PBS control group (ns, *p*> 0.05; *, *p*< 0.05; **, *p*< 0.01; ***, *p*< 0.001).





**Fig. S2.** J8-specific antibody response for BALB/C mice mice post-immunization with different sized J8-Lipo-DT (n=5/group). Mean antibody titers are represented as a bar + SEM. Statistical analysis was performed using a nonparametric, unpaired Mann-Whitney U test to compare test groups to the PBS control group (ns, *p*> 0.05; *, *p*< 0.05; **, *p*< 0.01; ***, *p*< 0.001).

**

**

**Fig. S3.** J8-specific antibody response for individual BALB/c mice (n=5/group) immunized with 7 weeks post-lyophilized J8-Lipo-DT. Mean antibody titers are represented as a bar + SEM. **A)** Salivary IgA titer. **B)** Serum IgG titer. Statistical analysis was performed using a nonparametric, unpaired Mann-Whitney U test to compare test groups to the PBS control group (ns, *p*> 0.05; *, *p*< 0.05; **, *p*< 0.01; ***, *p*< 0.001).
